# A Dual-Pronged Approach to Improving Heart Failure Outcomes: A Quality Improvement Project

**DOI:** 10.2196/13513

**Published:** 2020-02-10

**Authors:** Marcia Johansson, Ponrathi Athilingam

**Affiliations:** 1 College of Nursing University of South Florida Tampa, FL United States

**Keywords:** heart failure, mobile messaging, structured telephone support, self-care management, medication adherence, quality improvement

## Abstract

**Background:**

Presently, 6.5 million Americans are living with heart failure (HF). These patients are expected to follow a complex self-management regimen at home. Several demographic and psychosocial factors limit patients with HF in following the prescribed self-management recommendations at home. Poor self-care is associated with increased hospital readmissions. Under the Affordable Care Act, there are financial implications related to hospital readmissions for hospitals and programs such as the *Program of All-Inclusive Care for the Elderly* (PACE) in Pinellas County, Florida. Previous studies and systematic reviews demonstrated improvement in self-management and quality of life (QoL) in patients with HF with structured telephone support (STS) and SMS text messaging.

**Objective:**

This study aimed to evaluate the effects of STS and SMS on self-care, knowledge, medication adherence, and QoL of patients with HF.

**Methods:**

A prospective quality improvement project using a pre-post design was implemented. Data were collected at baseline, 30 days, and 3 months from 51 patients with HF who were enrolled in PACE in Pinellas County, Florida. All participants received STS and SMS for 30 days. The feasibility and sustained benefit of using STS and SMS was assessed at a 3-month follow-up.

**Results:**

A paired *t* test was used to compare the mean difference in HF outcomes at the baseline and 30-day follow-up, which demonstrated improved HF self-care maintenance (*t*_49_=0.66; *P*=.01), HF knowledge (*t*_49_=0.71; *P*=.01), medication adherence (*t*_49_=0.92; *P*=.01), and physical and mental health measured using Short-Form-12 (SF-12; *t*_49_=0.81; *P*=.01). The results also demonstrated the sustained benefit with improved HF self-care maintenance, self-care management, self-care confidence, knowledge, medication adherence, and physical and mental health (SF-12) at 3 months with *P*<.05 for all outcomes. Living status and social support had a strong correlation with HF outcomes. Younger participants (aged less than 65 years) performed extremely well compared with older adults.

**Conclusions:**

STS and SMS were feasible to use among PACE participants with sustained benefits at 3 months. Implementing STS and SMS may serve as viable options to improve HF outcomes. Improving outcomes with HF affects hospital systems and the agencies that monitor and provide care for outpatients and those in independent or assisted-living facilities. Investigating viable options and support for implementation will improve outcomes.

## Introduction

### Background and Significance

Heart failure (HF) is a clinical syndrome affecting 6.5 million Americans and is a growing problem around the world [1]. The gold standard of managing HF includes complex pharmacological, dietary, and device therapies that require self-management at home. Self-management can be defined as *daily activities that maintain clinical stability* [2]. For optimal health outcomes and stability, patients with HF must follow their prescribed self-management recommendations at home [3]. The most effective self-management strategies require patients to adhere to complex medication regimens, comply with diet and exercise recommendations, monitor symptom changes, and modify medications and behavior according to HF symptoms [4]. Individuals living with HF report significant negative effects on self-management at home with overall reduced quality of life; QoL [5]. In addition to the cost of human suffering, HF hospital readmissions are associated with over US $17 billion annually [6]. Up to 12.5% of these readmissions have been identified as preventable [7]. Several psychosocial and socioeconomic factors limit the adherence of patients with HF to self-management at home [8]. The current guidelines recommend telephone follow-up within 3 days and a follow-up visit within 7 to 14 days of hospital discharge [3]. The ultimate goal of follow-up care is to employ innovative approaches to keep people out of the hospital and at home [3]. Despite effective medical and symptom management strategies that are available, a considerable gap exists in the ability to effectively manage HF at home. Poor symptom management results in increased re-admission and affects individuals’ QoL [9]. A systematic review demonstrated that supporting people with HF at home using technology can reduce HF-related hospitalization and improve people’s QoL with improved knowledge on HF self-care [10].

Old age and multiple comorbidities challenge the ability of patients with HF to learn and continue self-management practices at home [11]. Many older adults live alone and lack social support, tending to rely on others such as visiting nurses and home care services. Many states offer home care nursing for people with chronic diseases leveraging the federal program known as the *Program of All-Inclusive Care for the Elderly* (PACE) [12]. PACE provides comprehensive medical and social services to frail, community-dwelling elderly individuals, most of whom are dually eligible for Medicare and Medicaid benefits. Individuals aged 55 years or older and living in the service area of a PACE organization are eligible for home care nursing. The goal of PACE is to promote older individuals to live safely in the community. Financing for the program is capped, which allows providers to deliver all services that participants need rather than limit them to those reimbursable under Medicare and Medicaid fee-for-service plans [9]. The PACE model of care is established as a provider in the Medicare program and enables individual states to provide PACE services to Medicaid beneficiaries [12]. However, under the Affordable Care Act, there are financial implications related to hospital readmissions for hospitals and programs such as PACE in Pinellas County, Florida.

Therefore, a prospective pre-post quality improvement project was conducted to assess the feasibility and preliminary efficacy of a nurse-led telephone support intervention supplemented with mobile phone SMS text messages in older adults with chronic HF who were enrolled in PACE. Previous studies have tested the effects of structured telephone support (STS) in patients with HF, but the use of SMS in this population remains underexplored. The project was completed at the Sun Coast PACE at Pinellas County, Florida. The project included participants with HF enrolled in PACE. A dual pronged STS with daily SMS was implemented to examine improvement in HF self-care, knowledge, and QoL.

### Rationale for Structured Telephone Support and Text Messaging

A systematic review of 49 qualitative studies found that much of the difficulty related to self-care management involved issues in remembering which self-care behaviors were appropriate or important to complete, as well as the harmful effects and perceived uncontrollability of HF symptoms [13]. A meta-analysis of 9 randomized clinical trials (RCTs) supported that individuals who received STS had a significantly lower risk of HF re-admission than controls (relative risk; RR 0.74; 95% CI 0.61 to 0.90) [14]. This is further supported by a review of 16 RCTs (n=5613) that exclusively implemented STS reduced HF-related hospitalization (RR 0.77, 95% CI 0.68 to 0.87; *P*<.01) [10]. A cost-effectiveness analysis of a 3-arm study, home visits with telephone calls (Home arm, n=196) and telephone calls only (Call arm, n=204), and control group that received standard care (Control arm, n=210) demonstrated that the telephone call arm had a higher probability of being cost-effective at 28 days and 84 days, whereas the home arm was less costly but less effective at 28 days and was dominating (less costly and more effective) at 84 days, indicating that a bundled intervention with home visits and telephone calls was less costly and more effective [15].

Given the increasing adoption and use of mobile technology, including text messaging in self-management of chronic diseases, a review on the use of mobile messaging for HF self-management was completed. Despite emerging evidence of mobile messaging use in several chronic conditions, the use of mobile messaging in HF has been limited. Among 60 patients with HF, daily messages from an interactive voice response system using an MP3 player with self-care management tips showed greater than a 50% reduction in the 30-day re-admission rate [16]. Similarly, a pre-post pilot study of patients with HF (n=15) reported that mobile messaging was easy to use 83% (12.5/15) and showed reduced pills missed 66% (10/15) and decreased salt intake 66% (10/15), with improved self-care maintenance (mean composite score increased from 49 to 78; *P*=.03) and self-care management (increased from 57 to 86; *P*=.02) at 4 weeks [17]. Mobile messaging was successfully used in cardiac rehabilitation [18], chronic health conditions [19], smoking cessation [20], weight loss [21], and medication adherence [22]. Therefore, the quality improvement project examined improvement in HF outcomes including self-care, medication adherence, and QoL after implementing STS with SMS among participants enrolled in PACE.

## Methods

### Overview

This project utilized a prospective pre-post design using a cohort of patients with HF enrolled in PACE in Pinellas County, Florida. Data were collected at baseline, 30 days, and 3 months. The sample included men and women with a clinical diagnosis of HF defined by the ICD-10-CM who were aged 55 years or above, a criterion for eligibility for PACE. The project was approved by the University’s institutional review board (IRB). Participants enrolled in PACE at Sun Coast Pinellas County, Florida were contacted via telephone and scheduled for a visit at the Sun Coast PACE day care center or in their homes. During the visit, the consent was reviewed with the participant and all questions were addressed and answered. A copy of the signed consent document was sent home with those participants who requested that their family be made aware of the study and necessary requirements. The participants were then consented using the IRB approved consent form and were not coerced to participate. Participants were provided with explanations about the STS and SMS program in addition to the care offered by PACE. A sample of the SMS used with participants included questions and encouragement of HF best practices (see Multimedia Appendix 1). Participants were informed that participation was voluntary for this project and the required follow-ups to be scheduled would occur at 30 days and 3 months.

### Intervention With Structured Telephone Support and Text Messaging

Once consented, all participants received STS 3 times a week over a 3-month period by an advanced registered nurse practitioner (APRN), who was a doctoral student. A standard protocol on delivering STS was developed to provide similar and consistent telephone support for all participants. Daily text messages on self-care tips on diet, exercise, HF symptom identification, and management as well as HF medications were also sent. Short messages on these topics with a total of 100 messages were developed and looped to be sent daily for 30 days. These messages were adapted for patients with HF and were similar to those utilized in studies using SMS for weight management. These messages were delivered automatically to participants’ mobile phone via a computer system. Of the 51 participants, 47 had mobile phones that could receive SMS and 4 participants were given a mobile phone to use during the study period. The phones that were given for use in the project had service set up through a national carrier to cover the 3-month study period. All participants were shown how to receive and read SMS with instruction and return demonstration by the APRN to assess ability to participate. A weighing scale and sphygmomanometer were also available to use if the participant did not own one. We measured the feasibility of STS and SMS among PACE participants by tracking the number of participants who completed the study at 30 days and 3 months. The sustained benefit from STS and SMS was assessed by participants’ scores at a 3-month follow-up.

### Measurements

Once consented, participants completed standard, validated outcome measures including HF self-care, knowledge, medication adherence, and physical and mental health. In addition, participants completed demographic variables and the social support questionnaire.

*Self-care behavior* was assessed using the valid and reliable Self-Care of Heart Failure Index that comprises 15 items with 3 subscales rated on a 4-point response scale [23]. Reliability of the Self-Care Maintenance subscale was *r*=0.56, Self-Care Management was *r*=0.70, and Self-Care Self-Confidence was *r*=0.82 [23]. Multiple studies have tested this scale on persons with HF [24,25].

*HF knowledge* was measured using the Atlanta Heart Failure Knowledge Test, a standardized validated instrument that is utilized both in research and clinical settings [26]. The question has 30 questions with a possible 0-30 score. Content validity ratings on relevance and clarity were tested in patients and family members that ranged from 0.55 to 1.0, with 81% of the items rated from 0.88 to 1.0. Cronbach alpha was .84 for patients and .75 for family members [26].

*Medication adherence* was assessed utilizing the 8-item self-administered Morisky Medication Adherence Questionnaire (MMAQ). The MMAQ has a Cronbach alpha of .83 [27] and demonstrated a sensitivity of 95% and a specificity of 53% at a cut-off point less than 6 and a total score of 10, and higher scores indicate worse adherence [27].

*Physical and mental health* was assessed using the SF-12 questionnaire [28]. The SF-12 was compared with SF-36 among cardiac participants and found to be valid with a correlation coefficient of physical component summary (PCS−12/−36; *r*=0.96; *P*<.001) and mental component summary (MCS−12/−36; *r*=0.96; *P*<.001) scores [29]. Similarly, change scores between baseline and 12 months were highly correlated (PCS−12/−36; *r*=0.94; *P*<.001) and (MCS−12/−36; *r*=0.95; *P*<.001). Therefore, to reduce patient burden, we used the SF-12.

*Demographic variables* included age, gender, race, and living status. Social support was assessed using the Duke-UNC *Functional Social Support Questionnaire* (FSSQ) short-version, which has 8 items in a 5-point Likert scale (1=much less than I would like and 5=as much as I would like) with internal consistency ranging from 0.50 for useful advice to 0.85 for help around the house [30]. The higher average score indicates greater perceived social support.

## Results

### Data Analysis

Data were analyzed using SPSS for Windows (version 21.0, SPSS, Inc). Descriptive statistics with frequency and percentage for categorical variables such as gender, race, and living status and mean and standard deviation for continuous variables were computed. Paired *t* test statistics were completed to compare baseline data with 30-days follow-up data to examine the effect of the intervention with STS and SMS with changes in mean score.

### Sample Characteristics

More than 100 records were reviewed to identify the patients with HF at PACE, and of those with the HF diagnosis, 85 were contacted for possible participation in the study over a 3-month period. A total of 51 eligible participants agreed and were enrolled in the study. Of those enrolled, about 90% (46/51) were aged 65 years and older, and the mean age was 77.39 (SD 9.34) years; 65% (33/51) were females. One-half of the participants (51%, 26/51) lived alone and 23% (12/51) lived in an independent or assisted-living facility. The other participants lived with a spouse or family member. About 71% (23/51) were white and 24% (12/51) were African American (see Table 1).

**Table 1:** Sample characteristics of the participants.

Characteristics		Value
**Age (years)**		
	Mean (SD)	77.39 (9.34)
	>65, n (%)	46 (90)
	Range	59-94
**Gender, n (%)**		
	Male	18 (35)
	Female	33 (65)
**Race, n (%)**		
	Hispanic	2 (4)
	Non-Hispanic white	23 (71)
	Non-Hispanic black	12 (24)
**Living status, n (%)**		
	Alone	26 (51)
	Independent or assisted living	12 (23)
	Lived with spouse or family member	13 (25)

### Feasibility of Using Structured Telephone Support and Text Messaging

Calculating the number of participants who completed the study at 30-days and 3-month follow-up assessed feasibility of using STS and SMS. All 51 participants (100%) completed the study at 30 days and 3-month follow-up indicating feasibility to use STS and SMS in this population, which could be utilized in designing and conducting a larger study.

### Potential Effect of Structured Telephone Support and Text Messaging on Heart Failure Outcomes

Owing to the sample size and lack of a control group, a dependent *t* test was used to compare baseline data with 30-day and 3-month follow-up data (see Table 2) to examine the effect of STS and SMS on HF outcomes. The results demonstrated that STS and SMS significantly improved HF self-care maintenance, self-care management, and self-care confidence and knowledge, medication adherence, and physical and mental health (Short-Form-12; SF-12) at 30 days and sustained improvement at 3 months with *P*<.05.

**Table 2:** Results of intervention at 3-month follow-up (n=51).

Outcomes	Baseline, mean (SD)	30-day follow-up, mean (SD)	3-month follow-up, mean (SD)	*t* test (*df*)	*P* value	Eta
Self-care maintenance	19.31 (3.61)	20.55 (3.13)	21.17 (2.92)	353.49 (49)	.01	0.935
Self-care management	9.67 (4.69)	10.16 (3.32)	22.72 (2.78)	438.24 (49)	.01	0.947
Self-care confidence	10.73 (3.70)	10.86 (3.57)	12.29 (2.85)	19.46 (49)	.01	0.443
Heart failure knowledge	23.98 (2.97)	27.82 (1.52)	28.84 (1.32)	91.19 (49)	.01	0.778
Medication adherence	3.14 (1.47)	2.84 (1.58)	2.78 (0.88)	6.84 (49)	.02	0.218
Short-Form-12	29.26 (2.88)	29.90 (2.90)	30.65 (2.41)	15.23 (49)	.01	0.383

### Factors That Potentially Influenced Heart Failure Outcomes

An analysis was performed to examine the effect of age, gender, race, living status, and social support on HF outcomes. HF knowledge was found to have a significant association with age (*F*_1,49_=4.01; *P*=.05; beta=.343; *R*^2^=0.376) indicating that those participants aged below 65 years had better HF knowledge.

Mental and physical health measured by SF-12 was also significantly associated with age (*F*_1,48_=5.47; *P*=.02; beta=.277; *R*^2^=0.328) indicating that younger patients with HF were less depressed than older patients. Medication adherence was associated with social support measured by FSSQ (*F*_1,49_=5.03; *P*=.03; beta=.305; *R*^2^=0.093). Those participants who lived with a spouse or family member had significantly improved self-care management (*F*_1,49_=−3.91; *P*=.01; beta=.456; *R*^2^=0.230). Living status also was significantly associated with HF knowledge (*F*_1,49_=6.52; *P*=.01; beta=.592; *R*^2^=0.343) and those who lived with a spouse or family member were less depressed as was evidenced by the SF-12 score (*F*_1,49_=16.47; *P*=.01 beta=.502; *R*^2^=0.252).

## Discussion

### Principal Findings

We conducted a quality improvement project that demonstrated that the dual pronged intervention with STS and SMS improved HF self-care, HF knowledge, and medication adherence and decreased depressive symptoms. The use of STS and the delivery of SMS served as a health care coach for this patient population, many of whom are isolated owing to age, living status, and symptomatic HF. Mobile technologies can augment health coaching by empowering patients and coaches to maintain numerous avenues for communication through voice and text message communication. A total of 90 email messages were sent to participants over the 3-month period (see Multimedia Appendix 1 for a sample of the messages). Participants were asked about the SMS and if they had received and understood the SMS. Questions were invited and requested during the STS communications.

Improvement in HF self-care was supported in a systematic review of 49 studies, which found that HF knowledge and self-care among HF participants, particularly regarding sodium reduction, medication adherence, weight monitoring, and physical activity, improved after STS [13]. These findings on improved self-care maintenance was also supported in a pre-post pilot study of patients with HF (n=15) that the text messaging improved self-care maintenance (mean composite score increased from 49 to 78; *P*=.03) and self-care management (mean composite score increased from 57 to 86; *P*=.02) at 4 weeks [17]. These findings are consistent with other research studies that supported improved medication adherence through the use of text messaging [31-33].

The findings also supported the assumption that living status and a higher level of social support had a strong correlation with HF self-care management and HF knowledge. The findings were supported in a longitudinal observational study of patients with HF (n=108) that a higher level of social support correlated with better outcomes in self-care behavior and adherence to medication, diet, and exercise over time [34]. These results were also consistent with those from a multisite trial conducted in the Netherlands (n=333) that showed a high level of social support was the only significant predictor for improved outcomes (beta=−2.65; 95% CI −4.45 to −0.85), as lower self-care scores reflect improved self-care [35].

The result of this project also indicated that those with good social support were less depressed (SF-12 score), and there was a strong association with depression and HF self-care [36]. Holzapfel et al [37] reported a significant association with self-care and age (*P*=<.05), which was also similar among our participants as those aged below 65 years fared better in HF knowledge and were less depressed compared with those aged above 65 years [37].

STS and SMS are feasible interventions among PACE participants. The result from this project indicated that all 51 participants (100%) completed the study at the 30-day and 3-month follow-up, indicating feasibility and acceptability by PACE participants. Sustained benefits from using STS and SMS were demonstrated at the 3-month follow-up.

The use of mobile phones has incorporated the ability to provide unparalleled care for patients with HF as it serves to maintain communication with participants and is convenient for use by the elderly with little coaching.

### Strength and Limitation

The study was completed at PACE, a member of Empath Health, a nonprofit integrated network of care supporting those challenged by chronic and advanced illness in the Tampa Bay region. Including participants enrolled at PACE served as a major strength and a limitation. PACE includes individuals above the age of 55 years, and thus 90% of the participants in the study were aged 65 years or older and 65% were females. PACE is an all-inclusive program, and patients with multiple physicians and obstacles with obtaining medications were not included along with a lack of generalizability to the overall population. Other limitations include the lack of a control group and use of self-reported measures and the outcome assessors and patients were not blinded. Hence, the possibility that the effects of the intervention were overestimated cannot be excluded. The small sample size was also a limitation; however, all study participants were able to complete the 3-month follow up.

### Conclusions

Implementation of STS and SMS was relatively easy to implement in this population. The costs incurred for this project were mainly related to providing participants a mobile phone with service, weighing scales, or sphygmomanometers if they did not own one. We are extremely thankful for the funding from the American Association of Colleges of Nursing (AACN) that supported this project. However, to be realistic, one must be cognizant of the lack of mobile phone availability for participants. Facilities such as PACE may need to tap into available resources to provide mobile service to the elderly who may live alone and educate them in using the service. HF symptom recognition and management are the mainstays of HF treatment, and interventions need to be tailored to improve self-care, knowledge, and QoL Sustained benefits with improved knowledge with STS and SMS need to be evaluated for a longer period. The results were disseminated to the PACE administrators for review and possible adoption of this model not only in the management of patients with HF but also of other clients with chronic diseases receiving care at that facility.

### Relevance to Clinical Practice

Nurses undertake many roles, including provision of direct care and clinical decision making. However, patient education and coaching are independent functions and standards in nursing care. On the basis of the results of our study, self-care management interventions involving STS and SMS could effectively improve various aspects for HF outcomes. It is important to educate nurses and patients with HF regarding the most common problems related to self-care of those with HF and the effective way to utilize STS and SMS. The results can also help the administrators of agencies such as PACE, home care agencies, and independent living facilities to incorporate self-management interventions with STS and SMS into patients’ daily treatment plan to prevent physical, psychological, and social problems that negatively affect patients’ ability to care for themselves.

## Appendix

Multimedia Appendix 1 Sample of SMS text messages.

## Abbreviations

AACN: American Association of Colleges of Nursing
APRN: advanced registered nurse practitioner
CDC: Centers for Disease Control and Prevention
FSSQ: Functional Social Support Questionnaire
HF: heart failure
IRB: institutional review board
MCS: mental component summary
MMAQ: Morisky Medication Adherence Questionnaire
PACE: Program of All-inclusive Care for the Elderly
PCS: physical component summary
RCT: randomized clinical trial
STS: structured telephone support
