# Supplementary material for: A Dual-Pronged Approach to Improving Heart Failure Outcomes: A Quality Improvement Project
Source: JMIR Aging. 2020 Feb 10;3(1):e13513. doi: 10.2196/13513 (PMC7055840; doi:10.2196/13513)
Supplement: Multimedia Appendix 1 [file aging_v3i1e13513_app1.pdf]

Sample of text messages sent to participants

1. Did you weigh yourself today?
2. Have you taken your blood pressure today?
3. Did you check your ankles for swelling today?
4. Are you following a low salt diet?
5. When eating out, ask for low salt items.
6. It is important to exercise for 30 minutes daily
7. Try to avoid being with others who may be ill and have flu like symptoms, like fever, cough, or body aches.
8. Heart failure is a problem in which the heart is unable to pump enough blood
9. Heart failure cannot be cured but it can be controlled
10. Try to stop or wean down the number of cigarettes you smoke in a day
11. Even if you feel better, DO NOT STOP taking your heart failure medications
12. People with heart failure take diuretics like Lasix. It is a water pill that helps remove excess fluid from your heart and lungs.
13. The best time of day to weigh yourself is upon waking in the morning.
14. If you notice that you have a weight gain of 2 to 5 lbs in 1-2 days, have increased swelling of the ankles and/or stomach, and have more shortness of breath, you should notify your doctor.
15. People with heart failure should exercise 2 to 3 times per week.
16. People with heart failure should weigh themselves every day.
17. People with heart failure who have a headache or pain should take Tylenol for relief of symptoms.
18. If you have been placed on a fluid restriction as part of your heart failure management plan and you feel thirsty, you can chew gum or suck on hard candy to help relieve this symptom.
19. If you forget to take your medication, you should take the medicine as soon as you remember.
20. It is important for someone with heart failure to see their heart failure doctor regularly.
